# Diagnostic Pathways and Genotyping of Cases of *Echinococcus granulosus* from Polish Patients

**DOI:** 10.3390/pathogens15050459

**Published:** 2026-04-23

**Authors:** Albert Gandurski, Marta Tokaj, Michał Jerzak, Aleksandra Popławska-Ferenc, Piotr Małkowski, Monika Dybicz

**Affiliations:** 1 Department of General Biology and Parasitology, Medical University of Warsaw, 02-004 Warsaw, Poland; s082426@student.wum.edu.pl (A.G.); s075324@student.wum.edu.pl (M.T.); 2 Department of General and Transplant Surgery, Medical University of Warsaw, 02-006 Warsaw, Poland; aleksandra.poplawska-ferenc@wum.edu.pl (A.P.-F.); piotr.malkowski@wum.edu.pl (P.M.); 3 Department of Surgical and Transplantation Nursing and Extracorporeal Treatment, Faculty of Health Sciences, Medical University of Warsaw, 02-006 Warsaw, Poland

**Keywords:** *Echinococcus granulosus*, cystic echinococcosis, polymerase chain reaction, molecular diagnostics, zoonoses, diagnostic pathways

## Abstract

Cystic echinococcosis (CE) is caused by a tapeworm of the *Echinococcus granulosus* s.l. species complex belonging to the *Taeniidae* family. CE affects more than 100 countries, including Poland, while remaining a significant public health threat to both humans and livestock. The aim of this study was to identify the genotypes responsible for cases of cystic echinococcosis in Poland by conducting molecular analysis of larvae isolated from Polish patients, and to investigate the diagnostic pathways leading to CE diagnosis. Between April 2023 and January 2025, tissue samples were collected from 10 patients following hepatectomy. Analysis of diagnostic pathways revealed that radiological findings followed by PCR or histopathological testing were sufficient to establish a reliable diagnosis of CE in 90% and 100% of cases, respectively. Serological tests showed lower sensitivity, reaching 86% for ELISA and 71% for Western blot. DNA extracted from all samples was used as the template in PCR to amplify and sequence the region of the mitochondrial NADH dehydrogenase 1 gene (nad1). PCR analysis confirmed presence of *Echinococcus granulosus* s.l. species in eight cases. All obtained nad1 sequences showed identity with the Echinococcus canadensis G7 (pig) strain. These results indicate that it remains the most frequent causative agent of human cystic echinococcosis in Poland.

## 1. Introduction

Cystic echinococcosis (CE) is a parasitic disease caused by the larval stage of the tapeworm belonging to *Echinococcus granulosus* sensu lato (s.l.) [1]. It is an important zoonotic disease with global distribution that affects more than 100 countries on every continent with the exception of Antarctica [2,3]. The highest prevalence has been reported in parts of Eurasia, northern and eastern Africa, Australia, and South America [4].

The global burden of CE remains substantial, causing significant economic losses and health problems in both rural and urban populations [2]. A recent study based on the Global Burden of Disease (GBD) 2021 database estimated CE mortality at 1364 (95% UI: 986–1776) deaths worldwide and attributed 105,071.6 (95% UI: 78,966.6–133,308.9) global Disability-Adjusted Life Years (DALYs) to the disease in 2021 [5].

In Poland, CE is not considered a frequent disease. According to the Polish National Institute of Public Health between 2014 and 2024 the number of reported human cases oscillated between 20 and 73 annually, as of 19 February 2025 [6,7,8,9,10,11,12,13,14,15,16]. Nevertheless, it remains expensive and complicated to treat and may require extensive surgery combined with prolonged drug therapy [17].

Usually, CE does not present clinical symptoms until cysts have reached a particular size. After a variable incubation period, growing cysts can exert pressure on adjacent tissues causing location-specific symptoms or rupture leading to systemic hypersensitivity reactions. In a considerable number of patients, CE is an incidental finding during routine imaging examinations. After initial suspicion further diagnosis is followed by a combination of imaging, serology, molecular, and histopathological testing [18]. Serological testing for *Echinococcus* antibodies is a common diagnostic method. However, a substantial number of patients do not elicit an immune response [19]. Ultrasonography (USG), magnetic resonance imaging (MRI) and to lesser extent computed tomography (CT) remain primary tools for detection and monitoring of hydatid cysts, particularly in seronegative cases. Nevertheless, atypical and varied imaging appearance depending on the parasite’s growth stage remains a major difficulty [17,20].

**Figure 1:**
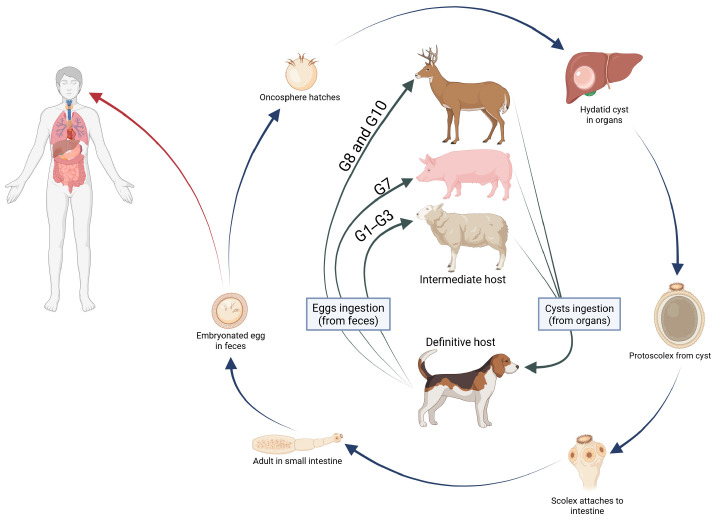
The life cycle of *Echinococcus* species.

The life cycle of *Echinococcus granulosus* s.l. is mostly domestic, involving dogs as the definitive host, in which the adult worm lives in the small intestine and releases infective eggs. Wild canids such as wolves and red foxes can also serve as definitive hosts in the transmission cycle. Intermediate hosts, which include sheep, swine, and cattle, together with humans as accidental intermediate hosts, acquire the infection through accidental ingestion of the tapeworm eggs. After ingestion of echinococcal eggs, oncosphere larvae are released and penetrate the intestinal lamina propria. Following penetration, the oncosphere is passively transported via the bloodstream to the liver in approximately 70% of cases, lungs in 20% of cases, or other internal organs where it develops into a hydatid cyst [21,22] (Figure 1).

The current taxonomic consensus divides *E. granulosus* s.l. into five different species: *E. granulosus* sensu stricto (s.s.; G1, G3), *E. equinus* (G4), *E. ortleppi* (G5), *E. canadensis* (G6–G8, G10), and *E. felidis*, though only the first four have been directly linked to human CE cases. It is important to note that there is an ongoing debate concerning the heterogeneity within the *E. canadensis* cluster, with propositions to subdivide it into three separate species: *E. intermedius* (G6/7), *E. borealis* (G8), and *E. canadensis* (G10). Nevertheless, for the sake of clarity, the more established classification featuring an undivided *E. canadensis* was utilized in this manuscript [23,24,25,26].

In Poland, the most commonly identified causative agent of CE is *E. canadensis* G7 genotype. Moreover, G7 has also been detected in human patients in Slovakia, Ukraine, Austria and Turkey [4,27,28,29,30].

The aim of this study was to identify the genotypes responsible for cases of CE in Poland by conducting molecular analysis of larvae isolated from Polish patients and to investigate diagnostic pathways that have led to CE diagnosis.

## 2. Materials and Methods

### 2.1. Materials

**Figure 2:**
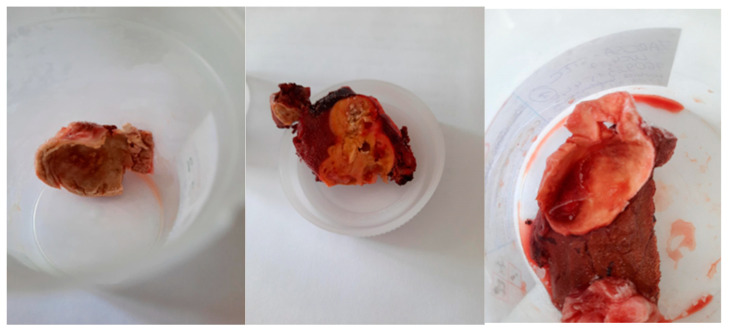
Representative liver tissue samples that underwent histopathological and molecular testing.

Between April 2023 and January 2025, tissue samples (Figure 2) were collected from 10 patients following hepatectomy at the Department of General, Transplant, and Liver Surgery, Medical University of Warsaw, Poland. Patient demographics, including age and gender, were recorded. Furthermore, imaging findings from USG, CT, and MRI were retrieved from the surgical department records.

Among the patients, 2 were male and 8 were female, with ages ranging from 33 to 70 years (Table 1). These patients were suspected of having CE. Nine out of ten patients underwent total pericystectomy, while the remaining one underwent partial pericystectomy (Table 1). The study was performed in accordance with the tenets of the Declaration of Helsinki.

### 2.2. Methods

**Figure 3:**
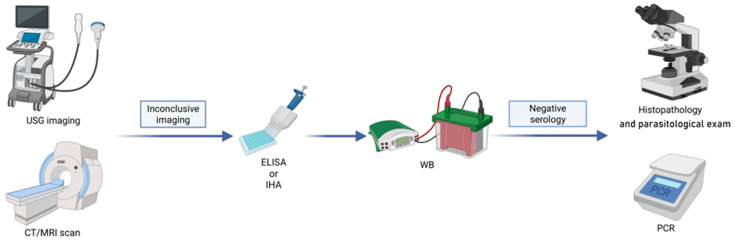
Diagnostic pathway.

Sera from patients with inconclusive imaging results were investigated by ELISA for the detection of IgG antibodies against *Echinococcus* spp. (Bordier Affinity Products S.A., Crissier, Switzerland) according to the manufacturer’s instructions [31,32,33] (Table 2). This procedure is routinely used as a screening test in our laboratory. Positive ELISA results were confirmed by Western blot (WB) using the LDBIO Diagnostics kit (Lyon, France). The subsequent step involved parasitological examination via light microscopy and PCR analysis (Figure 3).

The examined samples represented part of a cyst containing protoscoleces and/or hooks. The morphology of the fresh samples was studied directly. The wet unstained sediment of the cyst fluid and the inner layers of cyst tissue were examined for the presence of larva fertile elements by light microscopy (Olympus BX40, Olympus, Tokyo, Japan). The tissue samples, which consisted of parts of cysts, were either stored frozen at −20 °C or fixed in 70% ethanol prior to molecular analysis.

Tissue samples from the cyst wall were fixed in 10% buffered formalin, embedded in paraffin, and sectioned at 4–5 μm. The slides were stained with Hematoxylin and Eosin (H&E) and Periodic Acid–Schiff (PAS). The diagnosis was confirmed by identifying the characteristic laminated layer and the germinal membrane.

The tissue samples were rinsed several times with phosphate-buffered saline (PBS). The resulting suspension was then centrifuged at 3000× *g* for 10 min to collect the sediment. Each pellet was resuspended in 100 μL PBS, and genomic DNA was extracted using the NucleoSpin kit (Macherey-Nagel, Düren, Germany), following the manufacturer’s instructions.

A mitochondrial region was amplified by PCR using the extracted DNA as a template. A fragment of the NADH dehydrogenase 1 (nad1) gene was amplified with primers JB11 (5′-AGATTCGTAAGGGGCCTAATA-3′) and JB12 (5′-ACCACTAACTAATTCACTTTC-3′) [34,35].

Molecular identification was performed using a previously described PCR protocol [4]. The PCR reaction (50 μL) consisted of 1 μL of DNA template, 0.5 μM of each primer, 0.2 mM of each dNTP, 1× PCR buffer containing 2.5 mM MgCl_2_, and 2 U of Taq DNA polymerase (Qiagen, Hilden, Germany). Amplification was performed in a PTC-200 thermal cycler (MJ Research, Waltham, MA, USA) with the following conditions: 3 min at 95 °C, followed by 35 cycles of 1 min at 95 °C, 1 min at 55 °C, and 1 min at 72 °C [4].

The PCR products were separated by electrophoresis on 2% agarose gel (MetaPhor, FMC BioProducts, Philadelphia, PA, USA), stained with ethidium bromide, and visualized under a UV transilluminator. The nad1 gene fragments were then purified and directly sequenced in both directions using the BigDye Ready Reaction Cycle Sequencing kit and an ABI 3730 Genetic Analyzer (Applied Biosystems, Foster City, CA, USA). Sequencing was performed independently by three experienced laboratory technicians.

The resulting chromatograms were manually reviewed and edited using Chromas 2.0 software. The obtained sequences were aligned with others from the NCBI GenBank database using ClustalW2 [4,36].

## 3. Results

A total of 10 samples were obtained from patients who underwent surgery to remove a cyst located in the liver (8 cases of women and 2 men) (Table 1).

All 10 portions of tissue underwent histopathological examinations, and we assessed other diagnostic procedures in relation to their findings (Table 2).

All patients were examined by radiological techniques, with eight undergoing computed tomography and two magnetic resonance imaging. All radiology findings revealed cyst-like lesions of suspected parasitic origin. The cysts ranged in size from 12 mm to 60 mm in diameter, and the largest one was 6 cm (Table 1). One of the radiological examinations was false negative (Table 2).

Serology testing, which consisted of ELISA and Western blot tests, was performed for seven patients. Western blot was positive for four patients, while ELISA was positive for five. Only one ELISA result was a false negative, compared to two false negatives in WB analysis (Table 2).

DNA extracted from all 10 samples and positive controls was used as the template in separate PCRs to amplify a region of the mitochondrial NADH dehydrogenase 1. Each PCR produced a single band on agarose gel electrophoresis.

Eight out of ten isolates and controls were diagnosed as positive by amplification of nad1 fragment (~500 bp). The nad1 fragments were sequenced and compared with sequences of Echinococcus genotypes available in NCBI GenBank. One of the samples turned out to be a false negative. The sequences of eight isolates showed 100% identity with that of the pig strain G7, designated E. canadensis (Table 2). All nad1 sequences were deposited in GenBank with accession numbers PX121313–PX121320.

## 4. Discussion

In human infections in Europe, the most prevalent genotypes of *E. granulosus* s.l. are G1–G3 (sheep strains) as well as genotype G7 (pig strain). In addition, genotypes G8 and G10 (cervid strains) have been reported in Scandinavian countries and northern Europe [3,37].

Sheep strains are not only globally distributed but are also considered responsible for up to 88.5% of all human *E. granulosus* s.s. infections [25]. In Europe, locally acquired human infections have been reported in the United Kingdom, France, Poland, Ukraine, Austria, the Balkans, and most frequently in southern Europe, including Italy, Portugal, and Spain [3,4,25].

Although G7 accounts for approximately 11% of human CE cases worldwide, Casulli et al. demonstrated that this genotype is responsible for up to 28.3% of human CE cases in Europe [25]. This genotype has been identified in Germany, Austria, Poland, Lithuania, Slovakia, Romania, and Serbia [3,4,28,30,36].

There are several possible reasons why the G7 genotype is particularly prevalent in eastern and southeastern Europe. In rural Polish households, dogs commonly live in close proximity to livestock. Because canines play a key role in the transmission of *Echinococcus* spp., the prevalence of infection in dogs has a direct impact on the transmission, since it increases risk of human echinococcosis [38,39]. Although data on *E. granulosus* s.l. infection in dogs in Europe are limited, prevalence rates of up to 31% have been reported in Spain and Italy, and 14.2% in Lithuania, which borders Poland [40,41,42]. Another important factor contributing to the presence of the G7 genotype in Poland is infection in livestock, which represents a major source of hydatid cysts [3].

In Poland, all cases of human CE are subject to mandatory reporting. According to the Polish National Institute of Public Health, the number of reported cases in 2023 and 2024 was 66 and 73, respectively (status as of 19 February 2026) [15,16]. Although the number of cases included in this analysis was limited, they nevertheless represent a substantial proportion of all reported CE cases in Poland during the study period.

Among the analyzed patients, imaging followed by PCR and histopathological testing was sufficient to establish a reliable diagnosis of CE in 90% and 100% of cases, respectively (Table 2). Notably, CT and MRI examinations achieved a specificity of 90%, indicating high diagnostic value in this patient group. Despite the small study group, these findings highlight the significant value of imaging techniques in the initial stages of the diagnostic process, which remains consistent with current state of knowledge [43,44].

Serological tests demonstrated lower sensitivity, reaching 86% for ELISA and 71% for Western blot (Table 2). Despite this sensitivity, all patients required additional diagnostic procedures, including PCR and histopathology, to confirm the final diagnosis. Although serological tests remain convenient and non-invasive diagnostic tools that can be used in an ambulatory setting, they should be regarded as supplementary rather than definitive methods [45]. Clinicians should always bear in mind the false negativity of these tests. Cyst stage, localization (hepatic or extra-hepatic), size, number and integrity are known factors that may influence the results of serological tests to be false negative [45,46]. On the other hand, false positivity is also a serious issue that should not be overlooked when it comes to discussing serological tests in CE. A serious drawback of this method is cross-reactivity, which occurs during tapeworm or helminth infections, neoplastic diseases or liver cirrhosis [45,47]. Furthermore, false seropositivity may occur in endemic regions and it can be related to exposure to the parasite [45,48]. For these reasons, serology alone would perform poorly both as a definitive diagnosis method and as a screening tool. Our results highlight the auxiliary role of serological tests in the diagnostic process of CE and underscore the need for PCR and histopathology to confirm the diagnosis.

According to World Health Organization (WHO) guidelines on treatment of patients with CE, in developed countries surgery remains the standard of care for uncomplicated, active, multivesicular liver cysts that are bigger than 5 cm and for complicated liver cysts [17]. The two main types of surgical approach are total pericystectomy and partial cystectomy. Total pericystectomy is a radical approach that aims to resect the whole cyst, while partial cystectomy is a conservative approach that involves draining the cyst’s content and unroofing it [49]. Radical surgery has the best potential to completely cure the disease, since the whole cyst is removed from the patient’s body. Furthermore, the damage to healthy parenchyma is minimal. However, it should be kept in mind that radical surgeries create a great risk of intraoperative hemorrhages and dangerous cyst ruptures. On the other hand, conservative techniques are better options in complicated situations, such as communication with biliary tract. Unfortunately, the recurrence is significantly higher compared to the radical approach, as the remaining part of the cyst is a serious source of complications [50,51]. Surgeon should choose the preferred method according to patient’s condition, size and location of the cyst, available equipment and their own operating skills. Nine out of 10 of our patients underwent pericystectomy and only one underwent partial cystectomy.

Our findings are consistent with previous reports from this region, confirming that the G7 genotype remains the most prevalent genotype of *Echinococcus* spp. in Poland [4,36,52].

## Figures and Tables

**Table 1:** General and characteristics of patients and type of surgery.

Case Number	Sex	Age in Years	The Type of Surgery—Pericystectomy
1	M	33	Total
2	F	40	Total
3	M	41	Total
4	F	57	Partial
5	F	46	Total
6	F	56	Total
7	F	70	Total
8	F	61	Total
9	F	55	Total
10	F	56	Total

F Female, M Male.

**Table 2:** Results of diagnostic tests and procedures performed in patients during CE diagnosis and results of subsequent PCR analysis.

Case Number	Radiology Findings in the Liver (Cyst Localization and Size)	Serology ELISA WB		Parasitological Examination	Histopathology Diagnosis of Echinococcosis	PCR/Genotyping
1	MRI: segment 7/8; 59 × 43 mm	−	−	Hooks	+	Echinococcus canadensis (G7)
2	CT: segment 4b; 35 mm & segment 6/7	+	+	Lack of parasite elements	+	Echinococcus canadensis (G7)
3	CT: segment 7/6; 60 mm	+	−	Lack of parasite elements	+	Echinococcus canadensis (G7)
4	CT: segment 5/6/7/8	+	+	Lack of parasite elements	+	Echinococcus canadensis (G7)
5	CT: segment 6; 41 × 31 × 31 mm	+	+	Lack of parasite elements	+	Echinococcus canadensis (G7)
6	CT: segment 4/5 & segment 8/7; 30 mm	−	Not performed	Lack of parasite elements	+	Echinococcus canadensis (G7)
7	CT: liver hilum	+	−	Lack of parasite elements	−	−
8	MRI: segment 8; 35 mm & segment 6/7; 12 mm	+	Not performed	Lack of parasite elements	+	−
9	CT: segment 4; 30 mm & segment 7; 30 mm	+	Not performed	Larva and protoscoleces	+	Echinococcus canadensis (G7)
10	CT: segment 5/6/7/8	+	+	Lack of parasite elements	+	Echinococcus canadensis (G7)

WB, Western blot; ELISA, Enzyme-Linked Immunosorbent Assay; CT, computed tomography; MRI, magnetic resonance imaging; PCR, polymerase chain reaction.

## Data Availability

Publicly available epidemiological datasets analyzed in this study can be found on the National Institute of Public Health—National Institute of Hygiene (NIZP-PZH) website (https://www.pzh.gov.pl) and the World Health Organization website (https://www.who.int). The primary data obtained from the analysis of post-mortem samples are available on request from the corresponding author. These data are not publicly available due to ethical and privacy restrictions.
